# Arousing Effects of Electroacupuncture on the “Shuigou Point” in Rats with Disorder of Consciousness after Traumatic Brain Injury

**DOI:** 10.1155/2021/6611461

**Published:** 2021-04-19

**Authors:** Li Tan, Ning Wang, Zhilin Gu, Jiebin Zhu, Chunyan Liu, Zhenhua Xu

**Affiliations:** ^1^The Second Affiliated Hospital of Guangzhou University of Chinese Medicine, Guangzhou 510006, China; ^2^Shenzhen Longhua District Central Hospital, Shenzhen 518110, China; ^3^The Seventh Affiliated Hospital, Sun Yat-Sen University, Shenzhen 518107, China

## Abstract

Orexin is an important neuropeptide that stimulates cortical activation and arousal and is involved in the regulation of wakefulness and arousal. Our previous meta-analysis showed that acupuncture fared well in the treatment of TBI-induced DOC in which “shuigou (DU 26)” was the most important and frequent point targeted. In the present study, we investigated whether electroacupuncture (EA) promotes TBI-induced unconsciousness wakefulness via orexin pathway. A TBI rat model was established using a control cortical impact (CCI) model. In the stimulated group, TBI rats received EA (15 Hz, 1.0 mA, 15 min). In the antagonist group, TBI rats were intraperitoneally injected with the orexin receptor 1 (OX1R) antagonist SB334867 and received EA. Unconsciousness time was observed in each group after TBI, and electrocorticography (ECoG) was applied to detect rats' EEG activity. Immunohistochemistry, enzyme-linked immunosorbent assay, and western blot were used to assess the levels of orexin-1(OX1) and OX1R expression in the mPFC. We show that duration of unconsciousness and the ratio of delta power in ECoG in the EA group were significantly reduced compared with those in the TBI group. EA could increase OX1 and OX1R expression in the mPFC and reduced the loss of orexin-producing neurons in LHA. However, all the efficacy of EA was blocked by the OX1R antagonist SB334867. Our findings suggest that EA promotes the recovery of consciousness of TBI-induced unconscious rats via upregulation of OX1and OX1R expression in mPFC.

## 1. Introduction

Traumatic brain injury (TBI) is a global public health problem and a leading cause of death and disability [1]. According to recent studies, there were 27.08 million new cases and 55.50 million prevalent cases of TBI in 2016 [2]. The mortality rate of TBI in the US is 17.1 per 100,000 people compared with 12.99 per 100,000 people in China [3, 4]. Falls and road-traffic incidences are the main causes of TBI [5]. TBI is associated with intensive healthcare utilization, especially during the 1st year after injury. Therefore, TBI-related costs substantially burden families and society, with TBI-related costs estimated to be as high as £33 billion in Europe [6–8].

With the recent rapid development of intensive care technology and emergency medicine, the survival of patients with TBI has significantly improved [9]. However, these individuals also experience varying degrees of disorder of consciousness (DOC), which seriously affects their ability to perform activities of daily living and reduces their quality of life [10–12]. Early recovery of consciousness is closely associated with recovery in other functional domains, and the duration of DOC is an important prognostic factor in patients with TBI [13, 14]. The treatment for TBI-induced unconsciousness includes drug therapies [15], deep brain stimulation (DBS) [16, 17], hyperbaric oxygenation [18], and nerve electrical stimulation [19]. However, there is no specific drug for DOC, and DBS and electrical nerve stimulation are invasive treatments with certain risks and high surgical costs. Therefore, there is urgent need for effective, simple, and safe methods to promote awakening from an unconscious state. Our previous meta-analysis [20] showed that acupuncture fared well in the treatment of TBI-induced DOC in which “shuigou (DU 26)” was the most important and frequent point targeted. As such, acupuncture has been shown to be an effective therapy for TBI. In China, acupuncture has been used for centuries to promote recovery from DOC, considered to increase the supply of blood and oxygen to the traumatized area of the brain [21] and neuronal excitability [22, 23].

Orexin is a neuropeptide produced by the lateral hypothalamus (LHA) and is involved in regulating feeding behavior and the sleep-wake cycle. Orexinergic nerve fibers and orexin receptors are both found in the arousal systems, including the ascending reticular activation system, hypothalamus, thalamus, basal forebrain, and prefrontal cortex (PFC), which are regulated by the orexin system through orexin receptors [24, 25]. Furthermore, electrophysiological studies have revealed that orexin has a direct excitatory effect on cortical and cortical arousal-related neurons; therefore, it is believed that the orexin system may be the key source for the maintenance of arousal [26]. In addition, studies have shown that exogenous orexin can significantly prolong and enhance arousal under physiological conditions [27]. Clinical studies have shown that in patients in a coma, the orexin levels in the cerebrospinal fluid (CSF) after TBI are significantly lower than those in patients with normal consciousness [28].

Therefore, in the present study, we examined the effect of electroacupuncture (EA) on the behavior and electrocorticographic (ECoG) characteristics in rats with DOC induced by TBI. We then administered OX1R antagonists via intraperitoneal (i.p.) injection to examine whether the orexin pathway is involved in the awakening effect of EA. We further explored the expression of OX1 and OX1R in the medial PFC (mPFC) and the presence of orexin-positive neurons in the LHA.

## 2. Materials and Methods

### 2.1. Animals

All experiments were performed using 172 healthy Sprague–Dawley (SD) rats, weighing between 250 and 300 g. Rats were obtained from the Animal Experiment Center of the Guangzhou University of Chinese Medicine (Guangzhou, China). Twenty-eight rats were used for the ECoG recording experiment, and 144 were used for orexin validations. All rats were housed in a room maintained at 21 ± 2°C and were maintained on a 12-h light/dark cycle with free access to food and water.

All experiments were performed with the approval of the Animal Experimental Committee of the Guangzhou University of Chinese Medicine. All experimental procedures were conducted in accordance with the National Institutes of Health Guide for the Care and Use of Laboratory Animals (NIH Publication No. 85-23, revised 1985).

### 2.2. ECoG Electrode Implantation

Rats were anesthetized using isoflurane gas at concentrations of 4% and 2% in a vented anesthesia chamber. During the course of surgery, the rat's heart rate and respiration were monitored continuously. Under deep anesthesia, the rats were implanted with cortical EEG recording electrodes as previously described [29]. Briefly, electrodes were screwed through the skull on the dura over the mPFC (2.7–3.0 mm lateral to the midline, 0.7–1.0 mm anterior to the bregma), the reference electrodes were placed on the parietal cortex (3 mm lateral to the midline, 6 mm posterior to the bregma), and the earth electrode was placed on the cerebellar cortex (0 mm lateral to the midline, 10 mm posterior to the bregma) [30]. The rats were allowed to recover for at least 7 days after surgery; subsequently, we selected rats who had no neurological symptoms and their recording electrodes had not fallen off and randomly divided them into four groups: a control group (rats received sham surgery), TBI group (rats received TBI), EA group (rats received TBI and EA stimulation), and antagonist group (rats received antagonist injection, TBI, and EA stimulation), with six rats per group. The slow wave of EEG usually parallels the degree of consciousness and indicates its level. There is diffuse background rhythm from alpha (8–13 Hz) activity to theta (4–8 Hz) activity and subsequently delta (1–4 Hz) activity in the EEG, with a progression from lethargy to coma [31]. Therefore, in this study, we considered delta wave activity as a key indicator of unconsciousness.

### 2.3. TBI-Induced DOC Model Established

The TBI rat model was established using controlled cortical impact according to Smith et al. [32]. The 144 rats were assigned to four different groups (*n* = 36/group), and 12 rats were assigned to each time point (6, 12, and 24 h). In the control group, healthy rats received a sham operation and anesthesia. All rats were fasted 1 day before operation and were placed under isoflurane gas anesthesia at concentrations of 4% and 2% in a vented anesthesia chamber. Following deep anesthesia, the rats' head was fixed in a stereotactic apparatus (Reward, Shenzhen, China) and the skin covering the skull was incised. Body temperature was maintained at 37 ± 0.5°C using a heating blanket. After disinfection and following a midline incision, the soft tissues were reflected to expose the skull and anterior fontanelle. A 5 mm craniectomy was performed over the right parietal cortex to expose the dura mater, and then an impact rod was used to impact the dura mater with an impact speed of 3.5 m/ms, to a depth of 3 m, and with a residence time of 250 ms. After the blow, bone wax was used to close the bone window, and the scalp was sutured. Control sham injury rats were subjected to anesthesia and the surgical procedures but did not receive TBI. After each CCI or sham injury, the scalp was sutured, gas anesthetic administration was discontinued, and the righting time was monitored. Once ambulatory, the animals were returned to their home cages.

An animal was considered to have lost its righting reflex if it failed to correct its posture while lying on its back. Duration of loss of righting reflex (LORR) was evaluated by measuring the time interval between the appearance of LORR and recovery of the righting reflex after TBI as the criterion for the onset and termination of DOC.

### 2.4. OX1R Antagonist SB334867 Injection

SB334867, an OX1R inhibitor, was administered i.p., 30 min before unconsciousness induction. The inhibitor doses were modified from previous studies [33]. In the vehicle group, rats were injected i.p. with 10% dimethyl sulfoxide, 30 min before DOC induction.

### 2.5. Electroacupuncture

EA was performed after TBI and the first evaluation of consciousness. Rats in the EA and antagonist groups were treated with EA using an electric acupuncture apparatus (HANS-200, Suzhou, China). We opted for the “DU 26” acupoints according to our previous point analysis and research on acupuncture promoting awakening after TBI [34]. After local disinfection of the acupoints, an acupuncture needle was used for direct needling of 10 mm. In addition, a 1-cm needle was opened beside the nasolabial groove of rats, and another needle was used as a reference electrode. The electrode was connected to conduct EA (15 Hz, 1.0 mA, 15 min).

### 2.6. ECoG Recording System Connection

The recording wire was connected to the preamplifier of the recording system and then connected to the spike multichannel signal acquisition and processing system (CED, Cambridge, UK). Parameter settings were as follows: sampling frequency, 10000 Hz; high frequency filtering, 30 Hz; time constant, 0.2 s. ECoG was recorded every day from 19 : 00 to 19 : 00 the next day (i.e., 24 h).

**Figure 1:**
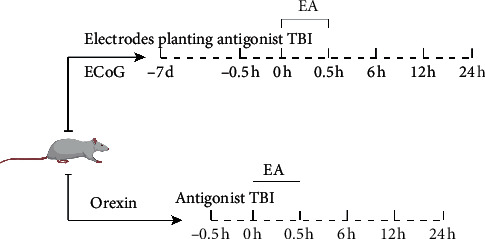
Experimental design.

The flow chart of the animal experiment design is shown in Figure 1.

### 2.7. Tissue Extraction

Rats in all groups were simultaneously euthanized with 10% pentobarbital sodium at 6, 12, and 24 h after TBI. Tissues from the mPFC and LHA were removed and analyzed with enzyme-linked immunosorbent assay (ELISA), immunohistochemistry (IHC), and western blot (WB) analysis to evaluate OX1 and OX1R expressions.

### 2.8. WB Assay

Six rats from each group were sacrificed at either 6, or 12, or 24 h after TBI by decapitation. The brains were carefully removed, and the mPFC was quickly dissected on ice. Protein was extracted by RIPA lysis buffer. The total amount of protein in each supernatant fraction was determined using the Pierce Quantitative Peroxide Assay Kit (Thermo, Waltham, MA), and an aliquot of each supernatant was removed and stored at −80°C. Homogenated samples containing equal amounts of protein and loading buffer were boiled for 5 min in water and run on a 10% sodium dodecyl sulfate/polyacrylamide gel. After electrophoresis, the proteins were transferred to polyvinylidene difluoride membranes. Membranes were blocked for 2 h at room temperature with TBS-T, containing 5% milk. The blots were incubated overnight with rabbit anti-OX1R polyclonal antibody (1 : 500, Ab68718; Abcam, Cambridge, UK) and rabbit anti-rat GAPDH monoclonal antibody (1 : 2000, Ab9485; Abcam) at 4°C overnight. After incubation, the membranes were washed three times with TBS-T and incubated for 2 h with horseradish peroxidase-conjugated goat anti-rabbit IgG (1 : 3000, Ab6013; Abcam) at room temperature for 1 h. The concentration of proteins was detected using a bicinchoninic acid protein assay. Then, blots were incubated with a chemiluminescence substrate and quantified using Quantity One software. WB analyses of OX1R in rat mPFC tissues were performed at 6, 12, and 24 h. The data generated are represented by optical density measurements of individual bands, normalized to the housekeeping protein GAPDH.

### 2.9. Enzyme-Linked Immunosorbent Assay

Prepared protein samples were tested using an ELISA kit for OX1 (CSB-088602; CUSABIO, Wuhan, China). After reagent preparation, wells for diluted standards, blanks, and samples were set up. The standards and samples were then diluted. When the reaction was completed, plates were read using a microplate reader (BioTek, Winooski, VT) and immediately measured at an optical density of 450 nm. The optical density of the standards was plotted against the log of concentration of the standards, and the concentration of OX1 was subsequently calculated.

### 2.10. Immunohistochemistry

The rats were anesthetized and decapitated at 6, 12, or 24 h after TBI and perfused through the heart with 4% paraformaldehyde. Next, the mPFC and LHA were carefully separated and fixed in 10% formaldehyde, dehydrated in graded ethanol, paraffin-embedded, and sectioned at 4 *μ*m coronal thickness for examination. Slides were baked at 65°C for 1 h after preheating followed by deparaffinization with xylene and rehydration. The slices were then placed in citrate buffer solution at high temperature for 20 min and then naturally cooled to room temperature. Afterward, the sections were treated with 0.3% hydrogen peroxide for 10 min, rinsed three times for 5 min each, and incubated with normal goat serum for 30 min. The sections were then incubated overnight at 4°C with rabbit anti-OX1R (1 : 250, Ab68718; Abcam) antibody and orexin antibody (1 : 250, Ab55051; Abcam). Following incubation, the tissue sections were rinsed extensively with PBS and then incubated with a biotinylated goat anti-rabbit antibody (1 : 2000, Ab6013; Abcam). Finally, the sections were reacted with diaminobenzidine and visualized under a florescence microscope (Nikon, Tokyo, Japan).

### 2.11. Statistical Analysis

The ECoG power was analyzed using NeuroExplorer (Plexon, Hong Kong). The positive IHC staining signals of OX1R in the mPFC and orexin in the LHA in a selected view were subjected to image analysis using Image Pro-Plus 6. The average gray value of WB was analyzed using Image J (National Institutes of Health, Bethesda, MD). All data were analyzed with SPSS 23.0 (IBM Corp., Armonk, NY). The mean ± standard deviation (SD) is used to express the measurement data in accordance with the normal distribution and homogeneity of variance. The differences between groups were analyzed with one-way analysis of variance (ANOVA), and pairwise comparisons were conducted with the Bonferroni method; the ratio data and those with non-normal distribution or variances were analyzed with nonparametric tests, and the median (*p*25–*p*75) is used to express these. A *p* value of <0.05 was considered statistically significant. All results were plotted using GraphPad Prism 8.

## 3. Results

### 3.1. Arousing Effects of EA on the Duration of DOC and ECoG of Rats with TBI-Induced DOC

**Figure 2:**
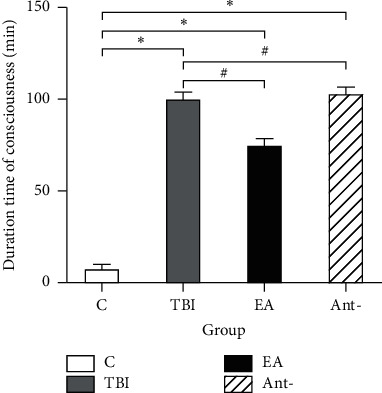
Effect of EA at “DU 26” on duration time of consciousness disorder in TBI rats. ^*∗*^*p* < 0.05, versus control group, and ^#^*p* < 0.05, versus TBI group.

The changes in behavior and ECoG of unconscious in TBI-induced unconscious rats were judged by the duration of unconsciousness after TBI and the comparison of the percentage of delta waves in ECoG at 6, 12, and 24 h after EA stimulation. As illustrated in Figure 2, as soon as the EA was applied to TBI rats, the recovery time was shortened to 75.33 ± 2.86 min, compared with that in the TBI group at 100.4 ± 3.13 min (*p* < 0.05) and in the antagonist group at 103.2 ± 3.13 min (*p* < 0.05). Meanwhile, there was no difference between the TBI and antagonist groups.

**Figure 3:**
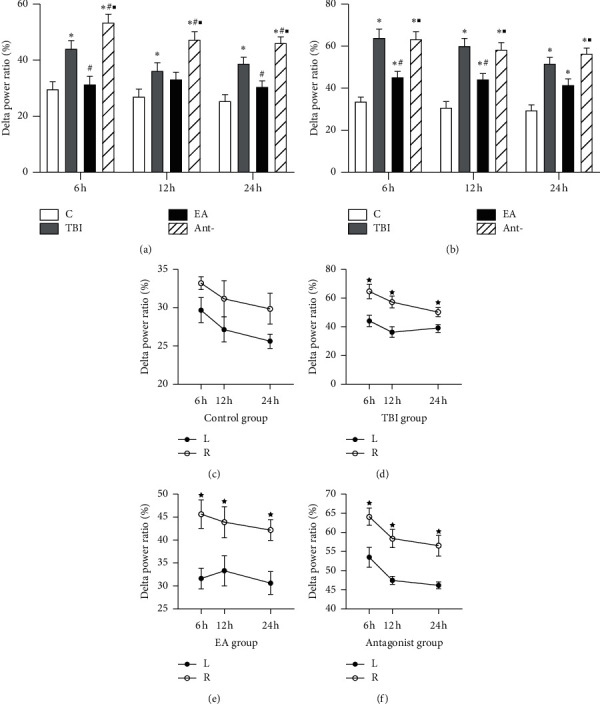
Effect of EA at “DU 26” on the delta power of ECoG in TBI-induced unconscious rats. Pooled data show the changes of delta power in ECoG after EA stimulation. (a): Delta power of ECoG on the left brain; (b): delta power of ECoG on the right brain (TBI side), (c): delta power of ECoG on the control group; (d) delta power of ECoG on the TBI group; (e): delta power of ECoG on the EA group; and (f): delta power of ECoG on the antagonist group.

As shown in Figure 3, the percentage of delta waves differed in the various groups at 6, 12, and 24 h, with the following trend: sham group < EA group < TBI group < antagonist group, suggesting that TBI could induce a decrease in delta power in ECoG. In the EA group, the delta power showed obvious changes compared with the TBI and antagonist groups. The delta waves in each group differed temporally, the left delta wave of the mPFC at 12 h was lower than that at 6 h, and there was no temporal difference between the left and right ECoGs in the sham group, but the proportion of the left delta wave in the TBI, EA, and antagonist groups was lower than that on the right side (*p* < 0.05).

### 3.2. EA Increased OX1 Expression in the mPFC of Rats with TBI-Induced DOC

**Figure 4:**
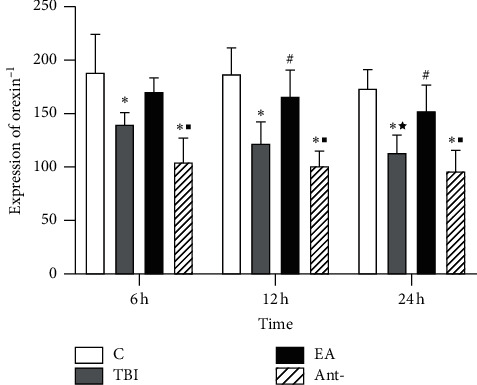
Effect of EA at “DU 26” on OX1 expression in the mPFC ELISA assay showed that EA treatment significantly increased OX1 level within the mPFC region 6, 12, and 24 hours after TBI. ^*∗*^*p* < 0.05, versus control group; ^#^*p* < 0.05, versus TBI group; and ^■^*p* < 0.05, versus EA group.

ELISA showed that the expression of OX1 in the TBI and antagonist groups was lower than that in the sham group (*p* < 0.05) at 6 h and lower in the TBI and antagonist groups than in the sham operation and EA groups at 12 and 24 h (*p* < 0.05) (Figure 4).

### 3.3. EA Increased OX1R Expression in the mPFC of Rats with TBI-Induced DOC

**Figure 5:**
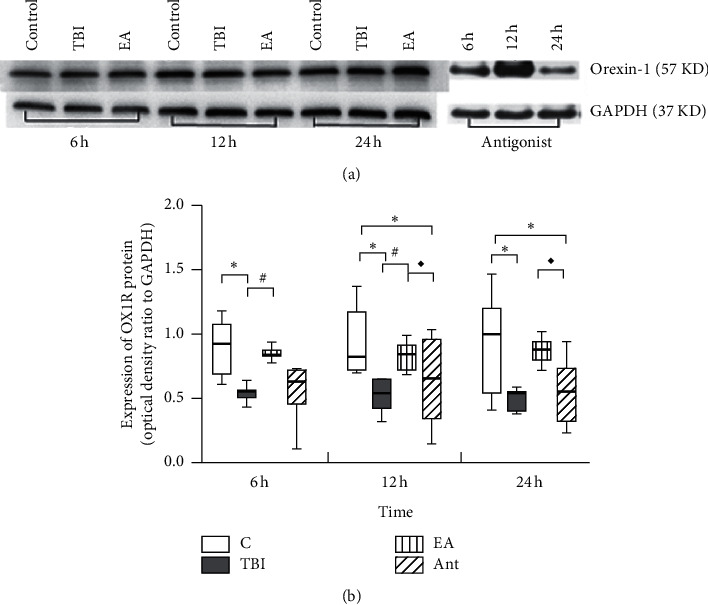
Effect of EA at “DU 26” on OX1R expression in the mPFC (western blot). ^*∗*^*p* < 0.05, versus control group; ^#^*p* < 0.05, versus TBI group; and ^■^*p* < 0.05, versus EA 3.4. EA reduced the loss of orexin-producing neurons.

**Figure 6:**
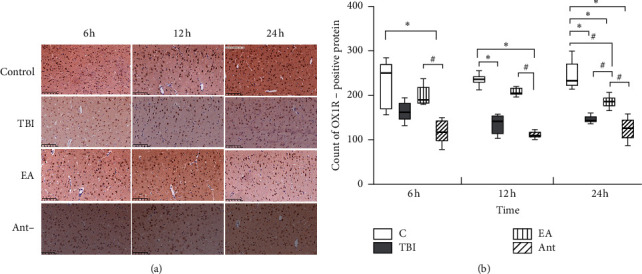
Effect of EA at “DU 26” on OX1R expression in the mPFC (immunostaining, 20 × 20). ^*∗*^*p* < 0.05, versus control group, and ^#^*p* < 0.05, versus EA group.

OX1R expression levels in the mPFC were measured using WB and IHC. In these two detection results, the expression level of OX1R in the mPFC in each group showed an increasing trend at 6 h, 12 h, and 24 h. OX1R expression was higher in the sham and EA groups than in the TBI and antagonist groups at all time points (Figures 5 and 6). There was no difference among the groups.

**Figure 7:**
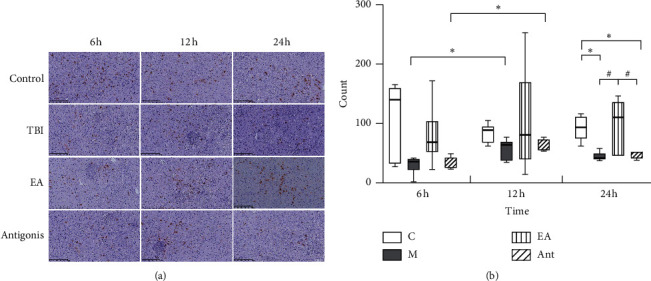
Effect of EA at “DU 26” on orexin-positive neurons in LHA (immunostaining, 10 × 10). ^*∗*^*p* < 0.05, versus control group, ^#^*p* < 0.05, versus EA group, and ^★^*p* < 0.05, versus 6 h.

Orexin-positive cells were present in all four groups, and the data showed that the number of orexin-positive neurons in the sham and EA groups was higher than that in the TBI and antagonist groups at 24 h (*p* < 0.05). Intragroup comparison of orexin-positive cells at 12 h in the TBI and antagonist groups was higher than that at 6 h (*p* < 0.05), and there was no significant difference in the other groups at different time points (Figure 7).

## 4. Discussion

The results of the current study mainly illustrated that EA can reduce the duration of unconsciousness and delta power in ECoG in DOC rats, subsequently exerting an arousal-promoting effect following unconsciousness induced by TBI. The arousing effect of EA on these unconscious rats may have been induced by the activation of OX1 and OX1R, resulting in the increase of excitability in the mPFC as shown in our study.

EEG can reflect the electrophysiological state of the brain by recording the postsynaptic potential of neurons in the cerebral cortex [35]. EEG is a sensitive indicator that reflects changes in brain function. It can be noninvasive, simple, and inexpensive and can provide bedside and real-time monitoring of large brain electrical activity [36]. EEG is one of the most commonly used neuroelectrophysiological monitoring and evaluation tools, which can reflect changes in the central nervous system function of patients with brain injury in a timely and accurate manner and can provide assessment of the awareness and prognosis of patients [37, 38]. Power spectrum analysis is commonly used as a frontal analysis method of EEG and can directly reflect the change in a patients' consciousness level. In ECoG, a recording electrode is implanted into the skull to record the electrical information and activity of brain neurons in a certain range near the electrode. Compared with the conventional scalp electrodes used to record EEG signals, ECoG electrodes are stable and less prone to outside interference and, as such, more suitable for animal EEG recordings. The percentage of the EEG power band in normal people is mainly the alpha frequency band. The EEG power spectrum of patients with consciousness disorder shows that slow wave (delta and theta) frequency bands increase and the degree of consciousness disturbance is directly proportional to the power value of the delta wave band [39]. Regarding the frequency distribution of the EEG power spectrum, it is generally believed that when the proportion of delta and theta wave bands increases, the excitability of the central nervous system decreases and vice versa [40]. Alster et al. found that the degree of disturbance of consciousness was closely related to the delta band of the EEG power spectrum [41]. Therefore, in this study, we analyzed the percentage of delta waves in ECoG to analyze recovery from DOC in rats. The results indicated that EA can shorten the duration of unconsciousness and reduce the percentage of delta waves in the ECoG power spectrum of TBI rats, which could improve the excitability of the cerebral cortex. However, after injection of the OX1R receptor antagonist, the arousing effect of EA on “DU 26” was inhibited, and this specific mechanism warrants further exploration.

Orexin, also known as hypocretin, is a small molecular neuropeptide that was discovered in 1998 and is mainly produced by specific neurons in the lateral hypothalamic area [42]. Orexin is an important neuropeptide that stimulates cortical activation and arousal and is involved in the regulation of wakefulness and arousal [43, 44]. Previous studies have shown that under physiological conditions, exogenous orexin can significantly prolong and enhance arousal. Similarly, selective activation of orexinergic neurons in the LHA can promote the transition from sleep to wakefulness in rats [26]. Using cardiac arrest to induce a comatose state in rats, Modi et al. [45] found that OX1 can promote awakening, reduce coma duration, and promote early functional recovery. Interestingly, Feng et al. [46] found that electrical stimulation of the median nerve can upregulate OX1 and OX1R in the LHA and promote the awakening of comatose rats after TBI. Additionally, the orexin antagonist, SB-334867, can block the awakening effect. Orexin can also promote arousal by regulating the expression levels of other neurotransmitters and related receptors, such as the 5-HT, NA, HT, and NMDA receptors [47–49]. Clinical studies [50–52] have shown that orexin levels in the CSF of patients in a coma after TBI are significantly lower than those in patients with a normal state of consciousness, and the mechanism may be related to injury of the thalamus and hypothalamus caused by trauma or other deep brain structure damage, ultimately affecting the orexin system. Baumann et al. [53] found that the number of orexin neurons in the hypothalamus after TBI was 29% lower than that in a normal group, assessed through pathological examination of four patients who died because of TBI. TBI caused loss of orexin neurons, which affected the recovery of consciousness in patients with consciousness disorder after TBI.

Orexin neurons project to almost the entire central nervous system and send strong excitatory projections to the brain areas involved in the regulation of cortical activation, such as the locus coeruleus, basal forebrain, nodular nucleus, and raphe nucleus, which promote the secretion of arousal excitatory neurotransmitters. Orexin specifically projects to the sixth layer of the cerebral cortex and widely activates the cerebral cortex through the effects of OX1R and OX1R receptors [53]. It has been found that the firing frequency of orexin neurons is negatively correlated with the delta band power of the EEG spectrum in the prefrontal cortex [54, 55]. Delta waves are mainly produced by thalamic cortical neurons [56, 57], which is one of the components of thalamic cortical concussion. Delta wave is the most common waveform of EEG in disturbance of consciousness. Jia et al. [27] found that OX1 can reduce the percentage of delta waves in the ECoG of alcohol-induced coma rats, increase the discharge frequency of frontal cortical neurons, and improve the excitability of cortical neurons, resulting in rat awakening. Previous studies have shown that orexin can reduce the proportion of delta waves in patients with TBI and promote their awakening [58]; however, the slow wave activity of EEG is significantly increased after selective inhibition of orexin neurons [59].

In the ECoG experiment, it was found that EA at “DU 26” could promote the recovery of consciousness and reduce the duration of consciousness and the proportion of delta waves, as well as reduce the loss of orexin neurons in the LHA and increase the expression of OX1 and OX1R in the mPFC region of unconscious rats after TBI.

Our study has some limitations that should be mentioned. First, we can use a single electrode or multichannel electrophysiological techniques to simultaneously record the direct effects of EA on orexin neurons in the LHA and in mPFC neurons. Additionally, we did not address whether EA directly acts on orexin neurons or whether it can indirectly excite orexin neurons through other intermediate neurons. Furthermore, there are alternative mechanisms worthy of further exploration. We only examined OX1 and OX1R expression in the mPFC, although other regions of the brain, such as the hypothalamus and brainstem, also participate in promoting wakefulness. Further studies are needed to clarify how the expression of OX1 and its receptor change following TBI-induced DOC and how EA causes increased OX1 expression. We also need to investigate the specific mechanisms and pathways underlying these changes. Moreover, it is well known that acupuncture often works through multiple targets; acupuncture may also activate other awakening pathways besides that of orexin. Furthermore, we did not use a nonacupoint control group to verify the effect of “DU 26” on promoting wakefulness.

## 5. Conclusion

In conclusion, EA can reduce the duration of unconsciousness and the proportion of delta waves as measured with ECoG, which could be a result of minimizing the loss of orexin neurons in the LHA and increasing the expression of OX1 and OX1R in the mPFC. Our findings suggest that EA is a promising method for awakening patients with TBI-related DOC. However, further studies are required to study the clinical effects of EA on “shuigou” and its possible mechanism.

## Data Availability

The data used to support the findings of this study are available from the corresponding author upon request.
